# New Trends in the Use of Volatile Compounds in Food Packaging

**DOI:** 10.3390/polym13071053

**Published:** 2021-03-27

**Authors:** Ana Beltrán Sanahuja, Arantzazu Valdés García

**Affiliations:** Analytical Chemistry, Nutrition and Food Science Department, University of Alicante, P.O. Box 99, E-03080 Alicante, Spain

**Keywords:** volatile compounds, active packaging, food packaging, antioxidant, antimicrobial, polymers, aroma compounds

## Abstract

In the last years, many of the research studies in the packaging industry have been focused on food active packaging in order to develop new materials capable of retaining the active agent in the polymeric matrix and controlling its release into food, which is not easy in many cases due to the high volatility of the chemical compounds, as well as their ease of diffusion within polymeric matrices. This review presents a complete revision of the studies that have been carried out on the incorporation of volatile compounds to food packaging applications. We provide an overview of the type of volatile compounds used in active food packaging and the most recent trends in the strategies used to incorporate them into different polymeric matrices. Moreover, a thorough discussion regarding the main factors affecting the retention capacity and controlled release of volatile compounds from active food packaging is presented.

## 1. Introduction

In the last years, many of the research studies in the packaging industry have been focused on food active packaging, in which the interaction between packaging and food is considered beneficial, in terms of positively modifying the sensory, nutritional, and microbiological properties of food, increasing its useful life and maintaining its quality [1]. In this sense, numerous studies have been carried out to develop new food packaging materials capable of retaining the active agent in the polymeric matrix and controlling its release into the food, which is not easy in many cases due to the high volatility of the chemical compounds, as well as their ease of diffusion within polymeric matrices [2,3].

Volatile compounds such as aldehydes, terpenes, and sulfur compounds, among others, are efficient active agents for a broad range of food applications due to their intrinsic antimicrobial, antioxidant, insect repellent, and other properties that can be suitable to extend the shelf-life of food that degrade under different mechanisms. However, they are also odorant and volatile at ambient temperature, properties that difficult their successful incorporation into a polymer matrix leading to high losses of the volatile compound and the formation of degradation products after processing and drying of the new developed materials [4]. In order to solve this limitation, in recent years, different processing methods have been designed and implemented apart from the conventional ones such as extrusion or injection molding, the main processing techniques used in the packaging industry in which high temperatures are needed causing losses of volatile compounds near to 30 wt% [5].

Among these techniques, the volatile compound can be introduced into the packaging as a coating layer directly applied on the material surface, as a self-supported film or as coated paper, when associated with a paper sheet. In addition, the encapsulation technology in which there is a carrier that entraps the volatile compound is a promising technique for the development of new materials with new specific functionalities such as controlled release of an active volatile principle, protection of substances against unstable and hostile media, masking of unwanted odors, immobilization, isolation of components in a mixture, and compatibilization, among others [6]. Regardless of the method chosen to prepare the capsules containing the volatile compound, a suitable encapsulation agent must be chosen, since it is important to take into account the final food application, the nature of all the components of the system, and its compatibility when processing.

Proteins such as whey protein isolate, sodium caseinate, soy protein, and zein, among others, are encapsulation matrices widely studied with volatile substances such as limonene that are encapsulated by spray drying or freezing [7]. Following this line, natural lipid molecules such as lecithin, cholesterol, liposomes, and nanoliposomes loaded with volatile chemical compounds such as eugenol have also been employed to develop materials with improved properties. Other biocompatible carriers such as emulsions and halloysites have been used as encapsulation systems for the incorporation of volatiles in food packaging materials [6]. In addition, cyclodextrins are materials also widely used in the encapsulation of volatile principles in packaging systems due to their high biocompatibility and the fact that they are materials approved by the FDA Food and Drug Administration (FDA) (Maryland, MD, USA). They can also form complexes with a large group of molecules, changing their chemical composition or biological behavior as they have been modified to improve their reactivity with the compound to be encapsulated. The incorporation of volatile compounds/cyclodextrin complexes in different matrices has been evaluated such as the addition of cyclodextrins containing carvacrol in coated papers [4].

However, since many of the encapsulates are introduced into the polymeric matrices using dissolution–evaporation systems (casting), being a not very practical method from the industrial point of view, alternative strategies have been developed such as electrospinning [6,8] This technique also allows for the improvement of the limitations presented by others such as spray drying due to the use of organic solvents that could generate toxicity problems in contact with food or aqueous solutions that lead to overheating of the capsules due to the need to raise the temperature to remove water and dry the final product [8]. In this sense, volatile compounds such as eugenol have been added to different polymeric matrices (polyvinyl pyrrolidone (PVP)/shellac fibrous film, poly(3-hydroxybutyrate) (PHB)/poly(3-hydroxybutyrate-co-3-hydroxyvalerate) (PHBV) film, gelatin nanofibers/poly(lactic acid) (PLA) film) by using electrospinning technique [9,10,11].

Acting after being released in the headspace without the need of a direct contact between the packaging material and the food it contains, the amount of the volatile compound has to be monitored and controlled at experimental conditions similar to the storage ones related to the selected food application. On the basis of this, some studies have focused on the evaluation of the ability of a volatile compound to be retained into the polymer matrix, and afterwards, be released under certain conditions in order to obtain the desired technological effect for which it has been added [12,13]. The knowledge of the matrix interactions with volatile compounds is helpful to predict their mobility once introduced in the polymer matrix, highlighting its importance in understanding the mechanisms of release in the headspace or in contact with food.

This review provides an overview of the type of volatile compounds used in active food packaging and the most recent trends on the strategies used to incorporate them into different polymeric matrices. Moreover, a thorough discussion regarding the main factors affecting the retention capacity and controlled release of volatile compounds from active food packaging is presented.

## 2. Volatile Chemical Compounds Used in Food Packaging

**Figure 1:**
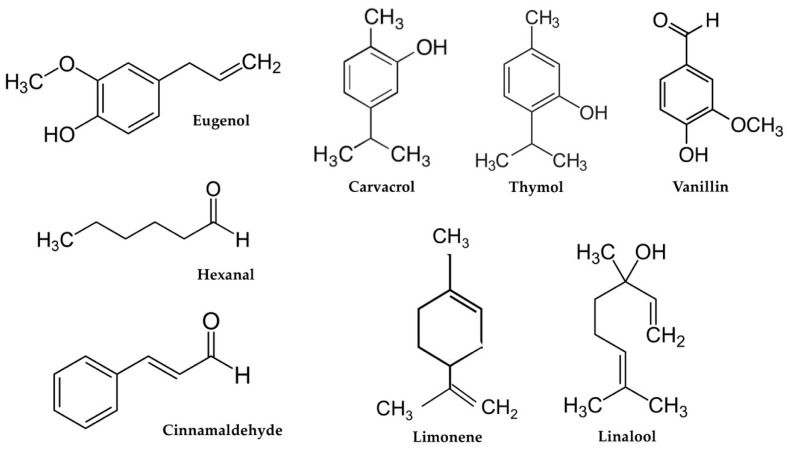
Chemical structure of the main volatile compounds recently used in active food packaging applications.

In recent years, a wide variety of volatile compounds has been reported for their use in food packaging. The incorporation of these chemical compounds in food packaging applications is continuously increasing because of changes in consumer demands as well as the new advances related to legislation and food safety. These substances mainly include chemicals such as terpenes, sulfur compounds, and aldehydes (Table 1). Chemical structures of main volatile compounds recently used in food packaging applications are shown in Figure 1.

### 2.1. Terpenes

The major volatile components used in food packaging found in recent literature are monoterpenes. All of them are obtained from essential oils (EOs), which are aromatic oily liquids obtained from plant material such as flowers, fruits, herbs, bark, roots, wood, buds, seeds, or leaves [24]. They are of great diversity, as they can consist of acyclic, monocyclic, or bicyclic structures and aromatic structural elements or unsaturated and saturated with different functional groups such as ketones, alcohols, or aldehydes [17]. By adding an oxygen functionality, the molecules are commonly called monoterpenoids. All of them are synthetized by many plants, with high interest for the food and perfume industries due to their aromatic properties.

Eugenol (4-allyl-2-methoxyphenol; C_10_H_12_O_2_), belonging to the allylbenzene class of phenylpropanoids, has potential analgesic, antioxidant, anti-inflammatory, and anti-cancer properties, being also efficient against microorganisms such as *Salmonella typhi*, *Escherichia coli*, *Salmonella paratyphoid*, *Bacillus licheniformis, Staphylococcus aureus*, and *Pseudomonas fluorescens* [25,26,27]. Chakraborty et al. [28] reported eugenol as the major compound of cinnamon leaf essential oil, whereas Hassan et al. [29] studied the antifungal activity of this compound as the main active volatile of clove oil. Eugenol is suitable for use in food packaging. In this context, it has been confirmed to show antioxidant and antibacterial activities and, also, the hydrophobicity of packaging [30]. However, the incorporation of eugenol to food packaging applications is usually limited by its high volatility and sensitivity to oxygen, light, and heat [31]. Nevertheless, eugenol has been used as active compound in food active packaging due to its antifungal [9], antimicrobial [10,32], and antioxidant properties [33].

Thymol (5-methyl-2-(propan-2-yl)phenol; C_10_H_14_O) and carvacrol (2-methyl-5-(propan-2-yl)phenol; C_10_H_14_O) are monoterpenes with well-known occurrence in thyme (*Thymus vulgaris* L.) and oregano (*Origanum vulgare*) essential oils. Thymol has a thyme-like and rosemary-like odor, whereas its structural isomer carvacrol shows an oregano, pencil, and wood odors [17]. For food applications, thymol and carvacrol are used as flavoring agents [34,35]. Moreover, both compounds have shown antimicrobial [36,37] and antioxidant [38] activities and act as safe food preservatives. Their incorporation in polymer films during processing also presents problems due to the sensitivity to temperature and also their high volatility [39].

Limonene (1-methyl-4-(prop-1-en-2-yl)cyclohex-1-ene; C_10_H_16_) is an important flavor component in lemon (*Citrus lemon*), making up over 95% of peel oil [40]. It is widely used as a food additive or flavoring agent and it has fungicidal properties, including activity against *Botrytis* and *Aspergilus niger* [41]. The main drawback of the use of this compound in active food packaging is its susceptibility of suffer oxidation processes [42]. In relation to the coriander, basil, and lavender essential oils, linalool (3,7-dimethyl-1,6-octadien-3-ol; C_10_H_18_O) is the main volatile compound present in their compositions [43]. It is noticeable its antimicrobial, anticancer, anti-inflammatory, analgesic, and anti-hyperlipidemic properties [44]. Unfortunately, as with other volatile compounds, linalool often presents an intense smell, continuous volatility, and poor water solubility [45]. Nevertheless, linalool has been used as active compound in food active packaging due to its antifungal, antimicrobial, and antioxidant properties. Carvone (2-methyl-5-(prop-1-en-2-yl)cyclohex-2-en-1-one; C_10_H_14_O) is a clear example of volatile compound where enantiomeric forms of the same compound can be isolated from different sources. In this sense, R-(−)-carvone (or l-carvone) can be found in spearmint with minty and sweetish medium-strength odor, whereas S-(+)-carvone (or d-carvone) is present in relevant amounts in caraway and dill with spicy aroma and medium strength [46]. In food science, d-carvone content is used as a flavoring to give a rye or caraway note, such as cheese, confectionery, sauces, bread preparations, and sauerkraut. On the other hand, l-carvone is used as a fruit flavor in bubblegum and beverages, with an improvement in sweetness and flavor by prolonging flavor duration. Moreover, carvone can be therefore mentioned as good antimicrobial agent to be used as active compound for food packaging applications [47,48].

Citral (3,7-dimethylocta-2,6-dienal; C_10_H_16_O) is the natural mixture of two acyclic aldehyde monoterpene isomers [49]: neral (*cis*-citral, β-citral) and geranial (*trans*-citral, α-citral). Both of them are found in the fruit and leaves of different plant species such as lemon, orange, tomato, and lemon grass [50]. Studies reported in the literature have suggested that citral has in vitro antimicrobial efficiency against *Listeria monocytogenes*, *Pseudomonas aeruginosa*, *Listeria innocua*, *Salmonella choleraesuis, Escherichia coli*, and *Staphylococcus aureus* [51], and thus its final functionality in food packaging is related to its antimicrobial activity.

Another volatile compound that has been incorporated into food packaging is p-cymene (1-methyl-4-(propan-2-yl)benzene; C_10_H_14_), which is found in more than 100 plant species, including many belonging to *Origanum*, *Thymus*, *Protium*, *Eucalyptus*, *Artemisia*, and *Ocimun* genera [52]. The essential oil of *Ciminum cymininunm* L. seed, which is mainly composed of p-cymene (47.08%), has been reported to show antifungal activity against *Aspergillus flavus* [53]. Moreover, antioxidant, antifungal, and antimicrobial properties have been underlined [54] that make it attractive for incorporation into active food packaging. Like other monoterpene hydrocarbons, γ-terpinene (4-methyl-1-(1-methylethyl)-1,4-cyclohexadiene; C_10_H_16_) is a stronger perturbator of cell membrane with moderately effectiveness as a bactericide against *Listeria monocytogenes*, *Streptococcus pyogenes*, and *Xanthomonas oryzae* [55]. This monoterpene is related to fruity notes [56]. For food packaging applications, this compound from oregano essential oil was introduced into a bioactive poly(lactic acid) (PLA) with polybutylene succinate (PBS) film extruded to protect products from spoilage [57].

On the other hand, valencene ((3R,4aS,5R)-4a,5-dimethyl-3-(prop-1-en-2-yl)-1,2,3,4,4a,5,6,7-octahydronaphthalene; C_15_H_24_) plays a fundamental role in citrus aroma, such as Valencia orange [58], with high odor thresholds, being a quality/maturity marker [59]. Thus, this volatile compound was used as a flavor agent in orange juices samples in glass bottles treated with different polymers such as low-density polyethylene (LDPE), polyethylene terephthalate (PET), and polycarbonate (PC) [60].

### 2.2. Sulfur Compounds

Diallyl disulfide (3-[(prop-2-en-1-yl)disulfanyl]prop-1-ene; C_6_H_10_S_2_) and allyl methyl sulfide (3-methylsulfanylprop-1-ene; C_4_H_8_S) are known as metabolic products of sulfur-containing foods, typically garlic (*Allium sativum*), and they are responsible for the characteristic pungent odor of garlic [61]. Garlic has been widely used as a flavoring agent with several pharmacological properties, such as antioxidant, anti-inflammatory, and antitumor capacities [62]. They are the major organosulfur compounds from garlic with similar biochemical activities [63]. Sulfur flavor compounds have high heat labile stability and strong pungent odors [64]. For food packaging applications, garlic essential oil shows antibacterial efficacy in the vapor phase against *Staphylococcus aureus* [65], being a promising alternative to be encapsulated in materials such as microcapsules to keep fresh the packaged food when not in contact with food.

Allyl isothiocyanate (3-isothiocyanatoprop-1-ene; C_4_H_5_NS) is originated from the hydrolytic cleavage of the glucosinolate by myrosinase. It has pungent and sulfur-containing aroma in cabbages and aroma of *Brassica* vegetables [66,67]. All of them are characterized by their high volatility. In terms of food packaging, it has been used as an antimicrobial agent encapsulated against *S. aureus* and *E. coli* [68].

### 2.3. Aldehydes

Vanillin (4-hidroxi-3-metoxibenzaldehido; C_8_H_8_O_3_) is a volatile compound with a distinctive odor that originates from vanilla beans, having strong antimicrobial activity when incorporated into beads, coatings, and film formulations [69,70]. This compound is one of the most widely used flavoring agents as a food preservative, in cosmetics, as a textile fragrance, and in dug preparations. In addition, its antimicrobial and antioxidant effect against molds, yeasts, and bacteria has been reported in the literature [71]. Cinnamaldehyde ((2E)-3-fenilprop-2-enal; C_9_H_8_O) is naturally found in cinnamon essential oil with strong antimicrobial effectiveness due to their phenolic structure and its ability to disturb the cell membrane of microorganisms [37].

Finally, aldehydes are chemical compounds composed by chain lengths of six and nine carbons such as hexanal (C_6_H_12_O), octanal (C_8_H_16_O), nonanal (C_9_H_18_O), and decanal (C_10_H_20_O). They have been shown to increase the shelf life of fruits and berries [72]. Thus, these compounds can be applied by the food industry to reduce the growth of fungi and bacteria [73]. Therefore, hexanal has been reported as antimicrobial agent [74,75].

## 3. Incorporation Strategies of Volatile Compounds into Food Packaging

Volatile components can either be coated or impregnated on the packaging materials or be incorporated (interspersed) into them. There are different techniques for incorporating volatile compounds in polymer matrices. In this sense, Table 2 summaries current applications of major volatile compounds used in food packaging, in particular eugenol and thymol. Afterwards, section continues with Table 3 in which current applications of minor volatile compounds used in food packaging were summarized. Also, the present section reviews the main strategies used to incorporate volatile compounds into polymer matrices focused on their advantages and drawbacks. 

### 3.1. Extrusion Technique

Regarding food applications of extrusion processing, bread packaged into PLA/poly(butylene-succinate-co-adipate) (PBSA) containing 3 and 6 wt% of thymol prepared using blow film extrusion technique showed the delay of visible growth of yeast and mold by 7 and 9 days, respectively, as compared to 6 days in the control film, which was attributed to the release of thymol in the package headspace [76]. The antifungal compounds thymol and R-(−)-carvone were incorporated into poly(lactic acid) (PLA)-based polymer at 10, 15, and 20% by weight by extrusion processing [77]. The authors underlined that thermal processing resulted in the loss of antifungal compounds (thymol and R-(−)-carvone) remaining in the films at each processing step. The final concentrations of the volatile compounds in the antifungal films were reduced to lower than 50% of initial concentration loaded. The same conclusion was achieved by Del Nobile et al. (2009) [78]. In this study, low-density polyethylene (LDPE), poly(lactic acid) (PLA), and polycaprolactone (PCL) containing 7%, 10%, and 15% (*w*/*w*) of thymol, respectively, were processed by extrusion processing at temperatures from 80 to 150 °C.

Regarding eugenol, Garrido-Miranda et al. [79] studied the mechanical and morphological properties of PHB–thermoplastic starch (TPS)-organically modified montmorillonite (OMMT) bio-nanocomposite adding eugenol at 3 wt% by melt intercalation in a co-rotating twin-screw extruder with temperature profiles of 100, 140, 160, and 150 °C. However, the active activity of the material was not studied, underlying that the addition of eugenol showed the highest elastic modulus of the bio-nanocomposites in contrast to the control. Other volatile compounds added into polymer matrices by extrusion processing have been carvacrol [80], allyl isothiocyanate [81], limonene [82], linalool [83], vanillin [84], and cinnamaldehyde [85]. Similar conclusions were reported with relation to high losses of volatile compounds as a result of the high processing temperatures.

### 3.2. Solvent Casting

Some authors have tried to study the effect of volatile compounds incorporation by the casting method. Following this line, cashew gum (CG) and gelatin (G) films, at a weight ratio of 5.0 CG/5.0 G and 10 wt% glycerol in relation to the total weight, were produced by incorporating ferulic acid (1 wt%) as a cross-linking agent and citral (10 wt%) from lemon grass essential oil for bread packaging [86]. The experimental packaging provided six additional days of preservation to packed bread in comparison with the 3 days for the bread in commercial packaging. The films were also effective in inhibiting mold growth on bread for 10 days, thus increasing its shelf life. On the other hand, the potential migration of citral (10 wt%) and its effect on the quality (color and texture) of coalho cheese packed with cellulose acetate film prepared by the casting method were evaluated [87]. The active film did not promote major changes in the overall texture of cheese, and the yellow color was enhanced during the 25 days. In this way, the cellulose acetate film was able to trap the citral compound on the surface of the cheese during the first 10 days followed by its migration to the cheese center over time up to 20th day, being an alternative for improving the quality of physical properties of artisanal food products. In addition, D-limonene (0.5 mL per gram of solution) was added into two different film formulations, emulsion gluten- and ι-carrageenans (Cs)-based films, by following the casting method [88]. Comparing Cs with gluten-emulsified film, the latter showed more interesting encapsulating properties since D-limonene was released gradually during the analysis time. In contrast, D-limonene did not show great affinity to Cs film, maybe due to its high hydrophobicity. In a different study [89], thymol (10 wt%) was added to PLA by the casting method and chicken meat was tightly packed with the developed films using sealing equipment. Results showed that samples packaged into active films showed their freshness physically after 15 days without any changes on texture, appearance, and odor when samples were compared with the control.

In this context, ultrasonic technology has been reported to enhance the ability of film molecules to obtain a dense film structure that allows for the addition of volatile compounds into edible films [90]. In this line, Fang et al. [91] prepared flaxseed gum films (1.5%, *w*/*v*) containing different contents of carvacrol (0.05, 0.1, and 0.2%) that were produced by a film-casting method with sonication with antioxidant and antibacterial activities. Different sonication powers (0, 400, and 600 W cm^−2^, respectively) and sonication times (0, 15, and 30 min, respectively) were applied. The results suggested that the addition of carvacrol could decrease the flaxseed gum molecular internal interactions, and sonication could result in a more homogeneous dispersion of carvacrol in the film matrix. As a result, the enrichment of flaxseed gum films with carvacrol ensured antibacterial properties against *S. aureus*, *Shewanella putrefaciens, Vibrio parahaemolyticus*, and *Pseudomonas fluorescens* bacteria. Moreover, the antioxidant properties of the developed films were improved. This phenomenon could be explained because the distribution of carvacrol was improved and a higher number of carvacrol molecules were exposed to the microorganism membrane.

Finally, the application of antibacterial films of yam starch with 1, 3, and 5 wt% of eugenol to pork preservation showed an antibacterial efficiency against *E. coli*, *S. aureus*, and *L. monocytogenes* attributed to the eugenol released from the films, which reacted with lipids and proteins and induced the destruction and denaturation of bacterial cell membrane to kill bacteria [31]. The application of starch film with 3% of eugenol was performed on pork preservation with reducing of bacterial colonies at day 7 of treatment in contrast to the control, which allowed the extension of the shelf life of pork beyond 50%. Fresh beef steaks were packaged onto oriented polypropylene film (OPP) coated with CA/S/eugenol or CA/eugenol coatings and monitored during a storage period of 14 days [30]. From this work, it is possible to conclude that the presence of eugenol preserved the red color characteristic of the sample whereas the lipid oxidation was hardly observed from 3 to 14 days of exposition, mainly attributed to the inhibition of gas-phase oxidation reactions with headspace free radicals and subsequent autooxidation in the food matrix.

### 3.3. Encapsulation

Encapsulation is defined as the process to entrap one substance (active agent) within another substance, yielding small particles that release their contents at controlled rates over prolonged periods of time and under specific conditions [92,93]. Different delivery systems have been developed to encapsulate bioactive volatile compounds in the food packaging sector such as cyclodextrins, halloysite nanotubes, emulsions, nanoparticles, covalent linkage with polymers, or core-shell nanofibers by coaxial electrospinning. Herein, we review the most used systems for food packaging development with emphasis in the novel strategies to introduce volatile compounds into different polymer matrices developed over the current years.

**Table 2:** Current applications of major volatile compounds used in food packaging. NA: not applicable.

Volatile Compound	Food Packaging	Processing Method	Concentration	Activity	Food Product	Reference
Eugenol	Polyvinyl pyrrolidone (PVP)/shellac fibrous film	Encapsulation followed by electrospinning	3, 6, 9, and 12% *w*/*w*	Antifungal	Strawberry	[9]
	Poly(3-hydroxybutyrate) (PHB)/poly(3-hydroxybutyrate-co-3-hydroxyvalerate) (PHBV) film	Electrospinning and annealing	2.5–25% *w*/*w*	Antimicrobial	NA	[10]
	Gelatin nanofibers/poly (lactic acid) (PLA) film	Electrospinning and annealing	2–4 mg g^−1^ gelatin	Antioxidant, antimicrobial	NA	[11]
	Poly(3-hydroxybutyrate) (PHB)-thermoplastic starch (TPS)/organically modified montmorillonite (OMMT) bionanocomposites	Extrusion and melt blending	3% *w*/*w*	NA	NA	[79]
	Starch film	Encapsulation followed by compression molding	1.2–1.6% *w*/*w*	Antioxidant	NA	[33]
	PHBV film	Encapsulation followed by electrospinning and annealing	2.5–20% *w*/*w*	Antimicrobial	NA	[32]
	Starch film	Casting technique	1, 3, and 5% *w*/*w*	Antimicrobial	Pork	[31]
	Cellulose acetate (CA) or acrylic component/hydrophobically modified starch (AC/S) coatings on corona-treated oriented polypropylene film (OPP)	Casting of CA on corona-treated commercial OPP	12.5 and 25% w/v	Antioxidant	Beef	[30]
Thymol	PLA/poly(ε-caprolactone) (PCL) blended films	Solvent casting method followed by supercritical CO_2_ impregnation of thymol	35.8% *w*/*w*	Antimicrobial	NA	[94]
	PLA/nanoclay C30B	Extrusion of PLA with organo-modified montmorillonite C30B followed by CO_2_ supercritical impregnation of thymol	17% *w*/*w*	Antimicrobial	NA	[95]
	PLA/PCL	Solvent casting method followed by CO_2_ impregnation of thymol	21.49% *w*/*w*	Antioxidant	NA	[96]
Thymol	Soybean protein isolate (SPI)	Solution casting method by adding thymol/diatomite complex	12.5% *w*/*w*	Antimicrobial	NA	[97]
	PLA/poly(butylene-succinate-co-adipate) (PBSA)	Blow film extrusion technique	3 and 6% *w*/*w*	Antifungal	Bread	[76]
	Starch/chitosan	Casting method followed by supercritical CO_2_ impregnation	27.3% *w*/*w*	Antimicrobial	NA	[98]
	PLA	Solvent casting	10% *w*/*w*	Antimicrobial	Chicken	[89]
	Poly(lactide-co-glycolide) (PLGA) nanofibers	Encapsulation of thymol in PLGA fiber via coaxial electrospinning	Encapsulation of 75% *w*/*w*	Antibacterial	Strawberry	[99]
	PLA	PLA extrusion followed by supercritical CO_2_ impregnation of thymol	20.5% *w*/*w*	NA	NA	[100]

#### 3.3.1. Electrospinning

Regarding the incorporation technique of the volatile compounds to the polymer matrix, special attention must be paid to the electrohydrodynamic (EHD) techniques such as electrospinning. In this line, Li, Dong, and Li [9] prepared PVP/shellac fibrous films by using a coaxial electrospinning technology at 30 °C and 40% relative humidity. The structural characterization of this novel material showed that a clear eugenol core–sheath structure at 3, 6, 9, and 12% *w*/*w* was obtained with moisture resistance and thermal stability up to 200 °C. Regarding the shelf life of packaged food, the weight loss, firmness, and pH of strawberries packed into PVP/shellac fibrous films/eugenol-loaded films was lower than that packed with PVP/shellac films with no obvious signs of decay for strawberries in PVP/shellac with 9% *w*/*w* and PVP/shellac with 12% *w*/*w* of eugenol on the sixth day. In the same line, different authors used the electrospinning encapsulation followed by annealing technique to introduce active volatile compounds into different polymer matrices such as PHB/PHBV [10], gelatin/PLA [11], PHBV films [32], and PLGA nanofibers [99].

Zhang et al. [99] encapsulated thymol (75 wt%) into PLGA to form core−shell nanofibers by coaxial electrospinning with an encapsulation efficiency of 90.3% to evaluate its feasibility as an antibacterial package for the preservation of strawberries. No obvious signs of decay for strawberries packed with the active nanofiber film were observed on the third day of storage. Thus, the developed film has a beneficial effect to prolong the shelf life of the studied fruit. Moreover, during storage, the growth rate of bacteria on strawberries coated with the thymol/PLGA nanofiber film was significantly lower in contrast to the control. In a different study, carvacrol (15% *w*/*w*) was encapsulated in polyvinyl alcohol (PVA) (15% *w*/*w*) matrices by electrospinning and casting [101]. The authors studied the effect of incorporating or not surfactant (Tween 85) in terms of encapsulation efficiency, thermal behavior, and microstructure by means of the electrospinning technique, in comparison with the casting technique. For the blend without surfactant, 83% of encapsulation efficiency was obtained. In contrast, 77% of encapsulation efficiency was obtained for the formulations, with surfactant being similar than that for the casting process. From a thermal study, it was possible to conclude that samples treated with the electrospinning technique without surfactant retained higher carvacrol fraction (40% of the total) in contrast to cast samples with a fraction accounting for about 18% of total carvacrol. Thus, the use of electrospinning as a delivery system was successfully applied for carvacrol-loaded PVA fibers in active packaging materials. Following this line, Jash and Lim [74] developed an hexanal precursor using a straightforward reaction between hexanal and *N*,*N*’-dibenzylethane-1,2-diamine to be added into two different polymer solutions [102]. On one side, the polymer solution was electrosprayed into ethylcellulose (EC) particulates or electrospun into PLA, with these techniques being suitable alternatives to control the release of hexanal from a permeable matrix, as is described in Section 4.

#### 3.3.2. Nanocarriers

The synergic effect of carvacrol essential oil and Montmorillonite (MMT) incorporated in thermoplastic starch (TPS) films in different contents (4.5, 9, and 15 wt%) has been reported in the literature [103]. Firstly, carvacrol (50 g) and MMT (25 g) hybrid were obtained by mixing with Tween 80 (10 g) and water (150 mL), followed by a drying process. Then, TPS films were obtained via casting method in which the hybrid was added to obtain the active material. Their physicochemical properties underlined that volatilization and degradation of carvacrol took place before the starch degradation but no quantification of the encapsulation efficiency and the release of carvacrol were studied. In a different study [104], carvacrol was microencapsulated by using β-cyclodextrin and incorporated to sodium alginate films at different concentrations (0, 15, 30, and 60 g L^−1^) obtained by solution casting to extend the shelf life of white mushrooms against *Trichoderma* sp. In this case, the encapsulation efficiency was obtained with a maximum of 89.65% when the core-to-wall ratio of microencapsulated powder was 1:10. Antifungal activity of films was evaluated by using the agar disk diffusion method for all the studied concentrations as a result of the antimicrobial effect of carvacrol that allowed for the destruction of the phospholipids, proteins, and lipids of the cell membrane. The best formulation was 30 g L^−1^ of carvacrol.

Covalent linkage has been examined by Arnon-Rips et al. [105]. In this case, hydrophobic vanillin (0.94 g dissolved in 2 mL ethanol, 6.2 mmol) was bounded to a positively charged chitosan (1.5% *w*/*v*) by Schiff base reaction and reductive amination applied as active coatings on fresh-cut melon. The coated fruit was stored for up to 14 days at 7 °C. To evaluate the effectiveness of coatings, we evaluated visual appearance by taking pictures of the fruit. After 8 days of storage, the uncoated fruit had a less appealing appearance, while fruit coated with chitosan–vanillin maintained good visual quality. Thus, it is considered that better antifungal was shown by the active coating. The covalent linkage allowed for overcoming the problem of lipophilic vanillin solubility without the need for an auxiliary encapsulating agent, and prevented the release of this volatile compound.

Regarding vanillin, one effective and challenging approach is the use of porous inorganic nanoparticles as nanoscale container to encapsulate active compounds and control their release. In particular, mesoporous silica and natural clay in tubular form are particularly attractive materials in terms of their controlling of the release of molecules embedded in the internal lumen by pore architecture, specific molecule/pore wall interactions, and pore size [106]. Stanzione et al. [13] designed a mesoporous silica-based delivery system by imine bond formation between amino groups and aldehydes group of vanillin. The amino group is provided by the aminofunctionalized mesoporous silica, which has been obtained through the functionalization of Santa Barbara Amorphous (SBA)-15 with aminopropyltrietoxysilane. The obtained particles have been embedded into PCL-based films by extrusion followed by melt blending in a hot press plate at 80 °C. In this work, it has been proven that the developed material exerts a significant influence on the release of vanillin from PCL polymer films as vanillin release profiles in water and ethanol has shown. In a different study [107], the essential oil of *Cinnamodendron dinisii* Schwanke, in which 1,8-cineole is the major volatile compound, was encapsulated in zein nanoparticles using the nanoprecipitation technique. The casting method was used to introduce these nanoparticles in order to obtain antimicrobial and antioxidant films. In this work, the developed packaging was applied in the conservation of ground beef. The encapsulation efficiency of the zein nanoparticles containing the essential oil was 99%, proving the proficiency of the nanoprecipitation technique. In samples where active films were used, meat samples showed lower production of malondialdehyde, suggesting that 1,8-cineole acted by stabilizing free radicals and the formation of reactive oxygen species, and, subsequently, decreasing the oxidative degradation of the lipids present in the ground meat.

The pronounced volatility and poor solubility of allyl isothiocyanate have limited this use. One solution reported in recent literature has been the use of halloysite nanotubes (HNTs), made of a natural aluminosilicate clay, to entrap it in a polymer network to maintain its stability, allowing its controlled release [68]. In this work, Maruthupandy and Seo encapsulated by vacuum pulling the allyl isothiocyanate volatile compound inside HNTs that were then coated with sodium polyacrylate (PA). PA is a polymer made up of long chains of negatively charged acrylic acid subunits and tightly bound sodium ions within a manacle of polyacrylate chains. Then, both ends and the surface of the HNTs were capped and coated by the PA polymer using an established surface layer coating method, obtaining a tube-like morphology. Novel developed materials were tested for antibacterial activity against representative Gram-negative (*E. coli*) and Gram-positive (*S. aureus*) bacteria by using the diffusion method at a concentration of 25 μg mL^−1^.

#### 3.3.3. Other Encapsulation Methodologies

Microencapsulation by spray-drying has been reported in the recent literature to encapsulate thermosensitive compounds since it is a fast process in which the temperature reached is relatively low. In this context, some authors microencapsulated eugenol by using the spray-drying methodology followed by a compression molding procedure [33]. In this case, soy lecithin, whey protein isolate, maltodextrin, and oleic acid were used to obtain microencapsulated eugenol with microcapsules with a mean diameter of around 15 μm. Films were obtained by mixing these microcapsules with starch and glycerol by melt-blending followed by compression-molding using a hot plate press at 150 °C. The eugenol content was determined by using the Folin-Ciocalteu method and through compound extraction and the measurement of UV absorbance. As a result, up to 45% of eugenol was retained into films.

### 3.4. Supercritical CO_2_ Impregnation

Regarding the incorporation methodology of thymol as volatile compound into polymer matrices, some studies suggested that the supercritical CO_2_ impregnation is an efficient technique for this purpose. The supercritical solution easily penetrates the polymer matrix due to the near-zero surface tension and low viscosity [108]. Different variables affect the impregnation efficiency. On one hand, the properties of the polymer, and on the other hand, the chemical interactions between the compound and the supercritical fluid. Finally, operational aspects must be considered, such as the depressurization rate and impregnation time. Milovanovic et al. [94] used an integrated supercritical extraction-impregnation process to isolate thyme extract and incorporate it into PLA/PCL blends. The film containing thymol (35.8 wt%) showed a strong antibacterial activity, leading to a total reduction of Gram-negative (*Escherichia coli*) and Gram-positive (*Bacillus subtilis*) model strains. Villegas et al. [95] developed a novel bionanocomposite on the basis of PLA reinforced with nanoclay C30B (5% *w*/*w*) impregnated with 1 g of thymol incorporated through supercritical impregnation using carbon dioxide obtaining up to 17% *w*/*w* of thymol impregnation. As a result, the new biomaterial was proven to be very effective since the viability of *E. coli* and *S. aureus* was not detected for both strains. In a different work, PLA/PCL blends with 21.49% of thymol loading improved flexibility, extensibility and ductility, and high antioxidant activity, with 124.06 ± 14.8 mg gallic acid per gram of film [96]. Pajnik et al. [98] reported the successful impregnation of thymol into starch-chitosan and starch-chitosan-zeolite (SCZ) films using supercritical carbon dioxide as a solvent. Moreover, a natural zeolite (15–60%) was used to increase the loading capacity of films when it was incorporated up to 27.3%. Films with the active compound (loading of approximately 24%) exhibited strong antibacterial activity against *S. aureus* and *E. coli*. Finally, thymol was incorporated into PLA films at concentrations up to 20.5% *w*/*w* by Torres et al. [100]. In this work, thymol was impregnated in PLA using the carbon dioxide technique. As a result, active biodegradable films were obtained to be used in a wide range of applications. As was observed for thymol, other volatile compounds have been added by using the supercritical CO_2_-assisted impregnation such as R(−)carvone to LDPE [109] and cinnamaldehyde to PLA [95], with impregnation yields of 4.38 and 13%, respectively.

**Table 3:** Current applications of minor volatile compounds used in food packaging. NA: not applicable.

Volatile Compound	Food Packaging	Processing Method	Concentration	Activity	Food Product	Reference
Carvacrol	Polyvinyl alcohol (PVA)	Electrospinning followed by casting method	15% *w*/*w*	NA	NA	[101]
	Flaxseed gum films	Solvent casting method by sonication	0.05, 0.1, and 0.2% *w*/*w*	Antioxidant and antimicrobial	NA	[91]
	Thermoplastic starch (TPS)	Montmorillonite encapsulation followed by casting method	4.5, 9, and 15% *w*/*w*	Antimicrobial	NA	[103]
	Sodium alginate	Encapsulation with β-cyclodextrin followed by solvent casting	15, 30, and 60 g L^−1^	Antifungal	Mushrooms	[104]
Vanillin	Chitosan	Covalent immobilization and casting method	6.2 mmol	Antimicrobial	Fresh-cut melon	[105]
	Xanthan gum-lignin hydrogel film	Hydrogel mixing followed by freeze-drying	0.9% *w*/*w*	Antimicrobial	NA	[110]
	PCL	Encapsulation in nanoparticles followed by extrusion and melting in a hot press	5 mL per gram of substrate	Antimicrobial	NA	[13]
Allyl isothiocyanate	Halloysite nanotubes (HNTs) coated with sodium polyacrylate (PA)	HNT encapsulation with PA by stirring and centrifugation	10 mg of HNTs per mL of allyl isothiocyanate oil	Antimicrobial	NA	[68]
1,8-Cineole	Chitosan	Nanoencapsulation and casting method	0.2% *w*/*w*	Antimicrobial and antioxidant	Ground beef meat	[107]
Citral	Cashew gum/gelatin	Casting method	10% *w*/*w*	Antimicrobial	Bread	[86]
	Cellulose acetate	Casting method	10% *w*/*w*	Improvement of physical properties	Coelho cheese	[87]
R-(−)-carvone	Low density polyethylene (LDPE)	Supercritical CO_2_-assisted impregnation	0.8 mg g^−1^ CO_2_	NA	NA	[109]
Cinnamaldehyde	PLA	Supercritical CO_2_-assisted impregnation	8 to 13% *w*/*w*	Antimicrobial	NA	[95]
Hexanal	PLA and ethylcellulose (EC)	Electrospinning and electrosprying	Hexanal into the polymer at 1:9 (*w*/*w*) ratio	Antimicrobial	NA	[74]
	Galactoglucomannans (GGM)	Hydrogel mixing followed by freeze-drying	1–100 mg g^−1^	Antimicrobial	Blueberries and cherry tomatoes	[75]
Octanal, nonanal, decanal, d-limonene, and eugenol	Epoxy, polyolefin, and acrylate can lining polymers	Empty cans exposition	4.1–4.2 ppb	NA	NA	[111]
d-Limonene	Gluten and ι-carrageenans	Casting method	0.5 mL per gram of solution	NA	NA	[88]

### 3.5. Others Technologies

Solid porous foams, such as aerogels, are lightweight materials that have a large surface area that may be used as delivery systems for active compounds, for example, in food packaging. In this line, aerogels based on polysaccharides such as galactoglucomannans (2 wt%) to release hexanal (1–100 mg g^−1^) [75] and xanthan gum-lignin (70/30 wt%) to release vanillin (0.9 mg) [110,111] have been developed by mixing followed by the freeze-drying process. In both studies, the antimicrobial activity of the developed materials was proved against different microorganisms. Moreover, the fungicide action of hexanal has been studied through the monitorization of hexanal release from galactoglucomannan aerogel to cherry tomato and blueberry samples. Levels of hexanal as low as 12–17 μmol L^−1^ in air were able to prevent softening of the studied cherry tomatoes, whereas the blueberries stored with hexanal-releasing aerogel remained visually unaltered for five days at room temperature in contrast to the control, in which the mold growth was evident. Thus, hexanal was proven to be useful for post-harvest preservation of fresh fruits.

**Figure 2:**
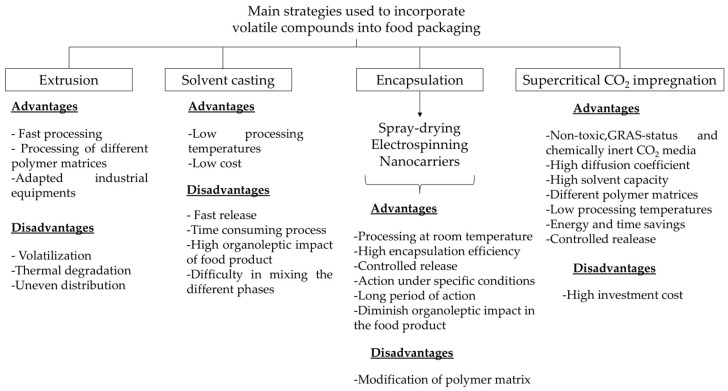
Scheme of advantages and disadvantages of main strategies used to incorporate volatile compounds into food packaging.

To summarize the present section, several parameters and properties must be taken into consideration to elucidate the most appropriate technique to incorporate volatile compounds into polymer matrices for food packaging applications. Figure 2 underlines advantages and drawbacks of the main methodologies currently used for this purpose.

Melting extrusion followed by compression molding is the most commonly used method in the packaging industry for the inclusion of active additives mainly due to the fact that industrial machines are adapted to this processing, working in a short time-period by using wide range of polymer matrices [95]. However, the application of this technique to incorporate volatile compounds is limited in the literature because the volatile agent can be volatilized or degraded due to the high temperature values usually used during the processing (higher than 145 °C). Moreover, uneven distribution is obtained when the volatile compound is added in the last minutes of the compounding process for preventing its evaporation. Thus, some authors have tried to study the effect of volatile compound incorporation by the casting method. This technique shows different advantages such as low cost and processing temperatures. However, compared with other methodologies such as encapsulation, casting allows for faster migration of the volatile compound due to the low penetration into the polymer with higher organoleptic impact in the food product [94] Moreover, it is a time-consuming process due to the drying phase with simultaneous evaporation of the volatile substance with the solvent, and sometimes it is difficult obtain the appropriate mixing of the system phases [91].

Encapsulation of volatile compounds solves some problems, for example, (a) increasing the solubility of compounds with an improvement of the compatibility with polymer matrices, (b) reducing losses due to high volatility or degradation during packaging manufacturing or storage, and/or (c) diminishing the organoleptic impact in food products caused by their strong odor [93]. As an example, microencapsulation by spray-drying is the most commonly used technique at the industrial level. It has been used to encapsulate thermosensitive compounds, since it is a fast process in which the temperature reached is relatively low. The wall materials in dried powder may also help to improve the retention of volatile compounds in the final encapsulates [33]. Electrospinning is one of the latest technologies to process volatile compounds into polymers in the form of monolayer and multilayer films by the application of high electric voltages and annealing treatments, having the main advantage of being able to operate at room temperature, obtaining high encapsulation efficiency [6,99]. As a result, volatile compounds can be directly incorporated in the core-shell morphology structure of the polymer, minimizing their volatilization or oxidation and reducing their release ratio [112]. Finally, a broad range of carriers have been developed to encapsulate volatile compounds in the food packaging, such as nanoclays, nanoparticles, cyclodextrins, covalent linkage, and halloysite nanotubes. However, one drawback that it could be related to the incorporation of encapsulated volatile compounds, for example by nanocarriers, could be the modification of the polymer matrix structure with changes in the functional properties. In this sense, Talón et al. [33] reported that the addition of encapsulated eugenol by spray-drying induced a heterogeneous film microstructure and caused notable changes in the tensile and barrier properties of the starch films. Thus, not all encapsulation systems can be applied in polymer matrices for food packaging as they should be compatible with the packaging material and do not negatively modify their barrier and mechanical and physical properties in order to preserve their primary function of food protection.

Finally, the supercritical CO_2_ impregnation uses a non-toxic, safe (Generally Recognized as Safe (GRAS) status), and chemically inert CO_2_ media as solvent to incorporate the active additive into the polymer matrix with high diffusion coefficient and solvent capacity in different matrices at a relatively low temperature. Its critical temperature is equal to 304.15 K, with lower energy demand being able to prevent the thermal degradation of volatile compounds [97]. During the process, the polymer exposed to CO_2_ undergoes swelling and the enhancement of chain mobility with CO_2_ plasticizing effect, which significantly accelerates the transport and facilitates incorporation of additive [96]. One drawback found for this technique is the CO_2_ plasticizing effect that could allow changes in the structural matrix of the polymer as Lukic et al. [96] reported for PLA loaded with thymol, in which lower tensile strength of the PLA film was obtained in contrast to the control. However, the main disadvantage is high investment cost compared to conventional processes because of high pressures applied (usually in the range from 10 to 30 MPa). However, operative costs are considerably lower due to the absence of the drying step and organic solvents [113]. Thus, this process has been proven to be an excellent method for loading of volatile compounds into polymer films, being a promising alternative for industrial applications.

## 4. Retention Capacity and Controlled Release of Volatile Compounds from Food Packaging

In relation to food packaging containing volatile compounds, the mechanism of action is based on the release of these compounds from the polymer matrix to the food sample to perform their technological function [4]. For this reason, the compatibility of the volatile compound with the polymer matrix and its capacity to be retained during processing are key parameters for the final validation of the active packaging for a specific food application. In addition, the mass transport processes through the packaging/food interface, such as release of volatile substances, must be controlled [12].

Among the variables involved in the retention and release mechanisms of volatile substances in packaging materials are the polymer nature and concentration; the volatile compound chemical structure and its concentration; the addition of fillers, plasticizers, and emulsifiers; the drying conditions; the relative humidity; and the temperature and the pH, among others [114]. On this basis, the development of food packaging containing volatile compounds depends also on the type of food that is going to be packed and the degradation processes it suffers during storage [6].

### 4.1. Retention Capacity of Volatile Compounds in Food Packaging

The retention capacity (%) of a volatile compound in a polymer matrix can be defined as the comparison of the amount of the volatile compound found in the packaging material after processing (m_f_; g volatile compound g^−1^ polymer matrix) and the theoretical content of the volatile compound initially added to the material (m_0_; g volatile compound g^−1^ polymer matrix) [4,33] under the following equation:

Retention capacity (%) = (m_f/_m_0_) × 100 (1)

When new food packaging materials are developed by the incorporation of volatile compounds, the retention capacity of these substances in the polymer is a key factor since higher losses of compounds are undesirable to avoid a negative impact in the final cost of the package and in the environment. In addition, a suitable amount of the volatile substance should remain in the material after processing in order to be able to perform the technological effect for which it has been added. The high volatility of these substances and the high temperatures often used in the packaging processing methods make this factor difficult to be controlled. Following this line, the retention capacity (%) of different volatile compounds incorporated to polymer matrices by using different processing techniques is discussed in this section.

In relation to the incorporation of carvacrol by using the casting method, its retention capacity was found to be higher in soy protein isolate (SPI) (95.3%) than in wheat gluten (WG) (85.5%) when increasing the carvacrol concentration from 15 to 30% *w*/*w*, indicating a suitable affinity between both components [4]. In relation to carvacrol/soybean protein isolate (SPI)-coated paper, the best drying experimental conditions were 250 °C for 20 s using an air dryer or a slow drying at low temperature such as 25 °C for 3 h at 50 ± 5% relative humidity (RH) in an oven. Due to the high volatility of this compound, the best drying conditions result from the application of short times (seconds) at high temperatures (>200 °C) or longer times (hours) but drying at room temperature. In another work, Higueras et al. [12] developed chitosan/cyclodextrin films containing carvacrol processed by casting that were stuck to the aluminum lid used to seal polypropylene/ ethylene vinyl alcohol/ polypropylene (PP/EVOH/PP) cups. The retention capacity of carvacrol in this packaging material was nearly 97% in comparison to the initial content, indicating that cyclodextrins exert a strong influence on the retention capacity of the volatile compounds in polymer matrices. On the other hand, when incorporating carvacrol into PP films by melt-blending followed by the compression-molding procedure [115], the authors reported low retention capacity (<50%), highlighting the strong influence of the temperature in this parameter.

Considering thymol, some studies have been focused on the incorporation of this compound into polymer matrices by using the supercritical impregnation technology. Torres et al. [100] evaluated the supercritical impregnation of thymol in polylactic acid (PLA), obtaining a higher impregnated amount of thymol (18–20%) when a lowest depressurization rate was used, indicating an increase in the interactions between the volatile compound and the polymeric matrix. The incorporation of zeolites as additives in the formulation have been proven to improve the retention of thymol in the polymer matrix. Pajnik et al. [98] studied the incorporation of thymol to starch-chitosan by means of the solvent impregnation technique using supercritical CO_2_. The impregnation values of thymol in starch-chitosan and starch-chitosan-zeolite films were compared, reporting an increase from 6.48–10.8% to 16.7–27.3% when the zeolite was added. These results indicate a high affinity of zeolite towards thymol that leads to suitable interactions between zeolite and phenolic groups of thymol, causing its retention in the matrix in higher amounts. Following this trend, the use of Ag nanoparticles to avoid losses of thymol incorporated into PLA by extrusion was evaluated by Ramos and coworkers [3]. The obtained value of the percentage of retained thymol in PLA when Ag nanoparticles were added to the formulation was about 76%, with this value being significantly higher than the value obtained when no Ag nanoparticles were added. Thus, the presence of Ag nanoparticles improved the retention of thymol in the polymer matrix, delaying the migration of the volatile compound through the polymer chains when the incorporation took place by extrusion. These results are in accordance with the ones obtained by Wu et al. [97], reporting that the addition of diatomite proved the reduction of losses of thymol incorporated to SPI, improving the retention capacity of thymol from 81% to 85.5% when adding this additive. This could have been due to the better intercalation of the diatomite containing the volatile compound in the polymer matrix in comparison with the addition of free thymol.

Following this line, Wicochea-Rodríguez et al. [4] studied the retention capacity of eugenol in SPI when it was incorporated by casting at 30 wt%, obtaining values of 75%. In addition, Valencia et al. [116] developed chitosan films containing eugenol encapsulated in lecithin liposomes, increasing the retention capacity from 1–2% when it was incorporated freely by emulsification to 40–50% when it was encapsulated prior to processing with chitosan. A similar effect was observed by Talón et al. [33], which reported higher values of retention capacity of eugenol in starch (S) films when adding the volatile compound encapsulated in whey protein (WP) or soy lecithin (LE) in the presence of oleic acid (OA). The obtained retention capacity values were 45 ± 4 and 45 ± 2% for eugenol-OA-WP-S and eugenol-OA-LE-S films, respectively, in comparison with a value of 26.6 ± 1.5% for retention capacity of free eugenol incorporated in starch by compression molding. These results proved the effect of OA as a carrier agent controlling the eugenol losses during processing, reducing its evaporation.

On the basis of the revised information, the processing technique employed to incorporate the volatile compound to the polymer matrix determines the retention capacity value of the compound. Values near to 30 wt% of losses related to volatile compounds incorporated to polymer matrices by extrusion methods are reported, and this loss is related to the high temperatures applied in the extrusion process [5]. In addition, the drying process also has an important impact in the retention capacity of a volatile compound into a polymer matrix since a progressive evaporation of the volatile compounds takes place during this process being affected by the applied temperature and time. Better drying conditions are short times (seconds) at high temperatures (>200 °C) or longer times (hours) but drying at room temperature. On the other hand, differences in the polymer type leads to differences in the dispersion of the volatile compound into the polymer chains and different molecular interactions conditioning the way the volatile compound interacts with the polymer. Finally, the presence of additives such as cyclodextrins, zeolites, nanoclays, montmorillonites, and liposomes, among others, in the formulation has also reported differences in the retention capacity of volatile compounds in the polymer matrix in comparison with formulations without these types of additives.

### 4.2. Release of Volatile Compounds from Food Packaging during Different Storage Conditions

Volatile compounds incorporated into polymer matrices can be released from the material to the headspace in the packaging/food system without direct contact between the packed food sample and the material. These compounds, due to their high volatility, can diffuse through the polymer matrix, reaching the packaged food, where can act on the food surface, or can be absorbed by the food sample. For this reason, the release of the volatile compounds from the packaging material to the food sample needs to be monitored and controlled in order to avoid higher volatile compound concentrations in food samples but assuring its functionality. In many cases, an increase in the release of the volatile compound is observed during the initial days followed by a stabilization [107].

**Figure 3:**
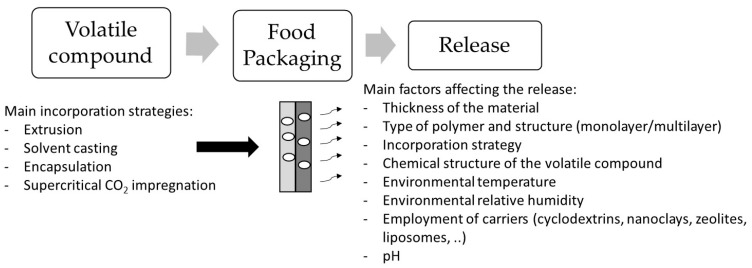
Scheme of the main factors affecting the release of volatile compounds from food packaging.

In addition to the interactions between the volatile compound and the polymer chains during the processing as mentioned above, several factors have a strong impact in the release of these substances from the packaging to the food sample. Among these factors, it is important to highlight the thickness of the material, the environmental temperature, relative humidity (RH), and pH, among others, that can induce structural changes on the polymer matrix, impacting in the release behavior of the volatile compounds [4] (Figure 3).

Due to the high volatility of the compounds and in order to assure that their release is avoided during the storage time of the packaging material but that it is activated once the food is packed, many works have focused on the use of polymers with volatile compounds entrapped in different carriers such as cyclodextrins, nanoclays, zeolites, etc., in order to control the release process.

Wicochea-Rodríguez et al. [4] described the release of carvacrol entrapped in cyclodextrins by using Avrami’s equation, obtaining a good prediction by modeling the carvacrol release with Fick’s second law. This equation was also used in other studies for evaluating the kinetics of active agents released from packaging materials [116]. It was reported that the release of carvacrol at 80% RH and 30 °C in SPI-coated paper was slower (77% of the initial content released in 1200 h) when it was added complexed with cyclodextrins in comparison with the behavior of free carvacrol (90% in 480 h). The effect of RH and temperature on the release of carvacrol from chitosan films was studied by Kurek et al. [114] by using the Fick’s second law to determine the diffusion coefficients. As the RH increased from 0% to >96%, a higher release was observed, which was attributed to the plasticization of the polymer matrix by water, increasing the interaction between chitosan and water and favoring the release of the volatile compound. The same trend has been reported in other work in which an increasing in the release of carvacrol incorporated by casting into SPI-coated paper was described when increasing the RH from 80 to 100%. [4] An increase in the release of carvacrol was also observed when increasing the temperature from 4 to 37 °C, indicating that the diffusion of the volatile compound is also accelerated when increasing the activation energy (Ea) [114]. In relation to the thickness of the films, it was reported that increasing the thickness of SPI films containing carvacrol from 27 to 133 µm led to a significant decrease in the release rate from 10.1 to 24.6 h^−1^ [4]. 

Following this line, Wu et al. [97] evaluated the effect of using diatomite to entrap thymol and then incorporated them onto a SPI film, observing a controlled and sustained release of the volatile compound from the polymer matrix to a food simulant solvent (60% glycerol) when diatomite was employed. This fact can be explained in that in the diatomite/thymol/polymer matrix system, the release of the volatile compound is not only affected by the diffusion coefficient of thymol in the polymer matrix but also by the diffusion of the volatile compound from the diatomite´s pores. In another work [99], thymol was encapsulated into poly(lactide-co-glycolide) (PLGA) to form core-shell nanofibers by coaxial electrospinning. The release of thymol was considerably reduced in comparison with the behavior of free added thymol, indicating a controlled release achieved using PLGA.

The release mechanism of vanillin was investigated by Stroescu and coworkers [117] in mono and multilayer materials of polyvinyl alcohol (PVA) and bacterial cellulose. The diffusion coefficients were calculated by using Fick´s law of diffusion, indicating a faster release of the volatile compounds in monolayer materials in comparison with multilayer films. In another work, Stanzione [13] and coworkers investigated the effect of mesoporous silica nanoparticles as carriers for controlling the release of vanillin incorporated into PCL composites by extrusion. The diffusion of vanillin from films containing the functionalized mesoporous silica was delayed by about 20% and 75% with respect to films containing free vanillin, in water and in ethanol, respectively. Considering eugenol, in order to evaluate the effect of pH, Talón and coworkers [33] studied the release behavior of free and encapsulated eugenol from chitosan films in simulants of different polarity and pH, such as ethanol 10% (*v*/*v*), acetic acid 3% (*v*/*v*), ethanol 20% (*v*/*v*), and ethanol 50% (*v*/*v*). By using Peleg’s empirical model, researchers have reported that the release ratio was accelerated in an acid medium, showing the effect of pH in the release behavior of volatile compounds that, in this case, could be attributed to the progressive partial hydrolysis of the starch matrix.

Alternatively, the release of hexanal can be controlled via precursor approach. Here, the volatile is released by selective cleavage of covalent bonds in the precursor compound by means of temperature, light, pH, or enzymatic/oxidative reactions [94]. Embedding the precursor in a permeable matrix would facilitate the release of hexanal from it being the electrospinning or electrospraying, a promising alternative for this purpose. In this line, Jash and Lim [74] developed an hexanal precursor using a straightforward reaction between hexanal and *N*,*N*´-dibenzylethane-1,2-diamine to be added into two different polymer solutions. On one side, the polymer solution was electrosprayed into EC particulates or electrospun into PLA. As was expected, release temperature and the carrier polymers affected the hexanal release rate as well as the maximal amount released.

On the basis of the revised information, we must highlight that there is not a general mathematical theory that can be applied to all types of different volatile substances when releasing from a packaging material. Different release behavior can be observed depending on the studied volatile compound, since different interactions can occur between the compound and the polymer matrix affecting the release rate and the mass transfer mechanism. Thus, the release of volatile compounds is also influenced by film composition, and a controlled release could be obtained using multilayered systems instead of monolayer systems in which the release rate is higher. The thickness of the materials also influences the release of volatile compounds, with the release being faster when the thickness decreases as a greater surface is exposed to the ambient. The RH and temperature are also important parameters affecting the release of a volatile compound from a polymer matrix, with many cases reporting an increase in the release rate as the RH and the temperature increase. However, it is important to highlight that the selected temperature and RH at which the release study is carried out depends, in many cases, on the conventional storage conditions of the selected food sample since it is advisable to carry out the release study in the real storage conditions (temperature, RH and time) of the selected packaged food. As an alternative, the encapsulation of the volatile compounds into different wall systems may limit its losses during film processing while also helping to modulate the release of the compound from the packaging material.

## 5. Legislation Remarks

As it has been observed, natural and organic compounds derived from vegetable and fruit sources have gained significant importance for food packaging applications for several reasons. They have a low cost and environmentally friendly nature, being the best alternative to commercial synthetic chemical compounds [118]. Moreover, the vast majority of compounds in Table 1 are obtained from EOs that have been registered by the European Commission (EU Reg. 1334/2008) for use as food flavorings [119,120]. In addition, compounds of Table 1 are classified as Generally Recognized as Safe (GRAS) by the U.S. Food and Drug Administration (FDA) [116]. Finally, hexanal is considered safe, and its use as a flavor substance is allowed in food products (EU No. 872/2012) [75]. Regarding the commission regulation (EU) No. 10/2011 and its actualization (EU No. 2019/1338) on plastic materials and articles intended to come into contact with food, only eugenol has been authorized to be used as a monomer or other starting substance that is chemically reacted to a macromolecular structure, the polymer, which forms the main structural component of the plastics.

## 6. Conclusions and Future Trends

The selection of the incorporation method and the concentrations of the active volatile compound into different polymer matrices can be determined by its thermostability as well as its action mechanism such as diffusion or volatility. In fact, different concentrations of the studied volatile compounds have been reported in the literature. Eugenol and thymol have been added up to 25–36% *w*/*w*, whereas other compounds such as vanillin and 1,8-cineole have been added in lower concentrations. One of the main drawbacks in the preparation of active packaging materials containing volatile compounds added by processing methods that implies high temperatures is related to the volatilization or degradation of the active components.

Once the volatile compound is incorporated into the packaging material, its retention capacity is difficult to elucidate since several factors have a strong impact on this parameter such as the selected processing method of the material, the chemical structure of the volatile compound and the polymer, the added amount of the volatile compound, the presence of other additives in the formulation (cyclodextrins, nanoclays, etc.), and the drying conditions. In addition, the release of the volatile compounds in polymer matrices not only depends on the interaction between the active compound and the polymer but also depends on the RH, time, temperature, pH, and thickness of the polymer material and on the presence of other additives able to protect the volatile compound, among others. A high retention capacity could be achieved for the selected volatile compound independently of the release pattern.

In general, further testing on in vivo real food systems under typical distribution and storage conditions would be essential to determine when and how amount of the volatile compound should be released to the food sample, a key factor in the development of food packaging prototypes.

## Figures and Tables

**Table 1:** Volatile chemical compounds commonly used in food packaging.

Volatile Compound	Chemical Class	Main Sources	Odor Quality	Reference
Eugenol	Monoterpene	Clove and cinnamon	Clove-like	[14]
Thymol	Monoterpene	Thyme	Thyme and rosemary	[14]
Carvacrol	Monoterpene	Oregano, thyme, and marjoram	Oregano, wood, and pencil-like	[15]
d-Limonene	Monoterpene	Citrus fruit peel	Lemon	[14]
Linalool	Monoterpene	Camphor tree and basil	Floral, sweet	[14]
R-(−)-carvone	Monoterpene	Caraway seeds, mint, and dill	Minty and caraway	[16]
Citral	Monoterpene	Lemon, orange, tomato, and lemongrass	Citrus and lemon	[16]
*p*-Cymene	Monoterpene	Thyme and horsemint	Wood and citrus	[17]
γ-Terpinene	Monoterpene	Variety of plants such as thyme	Turpentine-like and fruity odor	[18]
Valencene	Sesquiterpene	Citrus, mainly orange	Citrus	[19]
1,8-Cineole	Monoterpene	*Cinnamondendon dinissi* leaves and eucalyptus oil	Minty and herbal notes	[20]
Allyl sulfide	Sulfur compound	Garlic	Garlic	[20]
Diallyl disulfide	Sulfur compound	Garlic	Garlic	[20]
Allyl isothiocyanate	Sulfur compound	Cruciferous vegetables and black mustard seeds	Mustard-like odor	[21]
Vanillin	Phenolic aldehyde	Bean or pod of tropical vanilla orchid	Vanilla, sweet	[22]
Cinnamaldehyde	Aldehyde	Cinnamon tree	Cinnamon	[23]
Hexanal	Aldehyde	Edible oils such as sunflower	Green, grassy, soapy	[15]
Octanal	Aldehyde	Citrus oils	Green, citrus, orange peel	[16]
Nonanal	Aldehyde	Natural oils	Citrus, soapy	[16]
Decanal	Aldehyde	Citrus, buckwheat, and coriander essential oil	Green, citrus, fatty	[16]

## Data Availability

The data presented in this study are available on request from the corresponding author.
